# Adaptive response of Pseudomonas aeruginosa under serial ciprofloxacin exposure

**DOI:** 10.1099/mic.0.001443

**Published:** 2024-04-03

**Authors:** Thuc Quyen Huynh, Nguyen Bao Vy Tran, Thi Thuy Vy Pham, Vo Bao Tran Le, Thien Phu Truong, Van An Huynh, Thi Hang Tong, Thi Truc Ly Trinh, Van Dung Nguyen, Le Nhat Minh Pham, Thi Hiep Nguyen, Qifeng Lin, Teck Kwang Lim, Qingsong Lin, Thi Thu Hoai Nguyen

**Affiliations:** 1 Research Center for Infectious Diseases, International University, Ho Chi Minh City, Vietnam; 2 School of Biotechnology, International University, Ho Chi Minh City, Vietnam; 3 Viet Nam National University, Ho Chi Minh City, Vietnam; 4 Cho Ray Hospital, Ho Chi Minh City, Vietnam; 5 Gia Dinh People’s Hospital, Ho Chi Minh City, Vietnam; 6 School of Biomedical Engineering, International University, Ho Chi Minh City, Vietnam; 7 Protein and Proteomics Centre, Department of Biological Sciences, National University of Singapore, Singapore, Singapore

**Keywords:** antibiotic resistance, ciprofloxacin, iTraq labelling, proteomics, *Pseudomonas aeruginosa*

## Abstract

Understanding the evolution of antibiotic resistance is important for combating drug-resistant bacteria. In this work, we investigated the adaptive response of *Pseudomonas aeruginosa* to ciprofloxacin. Ciprofloxacin-susceptible *P. aeruginosa* ATCC 9027, CIP-E1 (*P. aeruginosa* ATCC 9027 exposed to ciprofloxacin for 14 days) and CIP-E2 (CIP-E1 cultured in antibiotic-free broth for 10 days) were compared. Phenotypic responses including cell morphology, antibiotic susceptibility, and production of pyoverdine, pyocyanin and rhamnolipid were assessed. Proteomic responses were evaluated using comparative iTRAQ labelling LC-MS/MS to identify differentially expressed proteins (DEPs). Expression of associated genes coding for notable DEPs and their related regulatory genes were checked using quantitative reverse transcriptase PCR. CIP-E1 displayed a heterogeneous morphology, featuring both filamentous cells and cells with reduced length and width. By contrast, although filaments were not present, CIP-E2 still exhibited size reduction. Considering the MIC values, ciprofloxacin-exposed strains developed resistance to fluoroquinolone antibiotics but maintained susceptibility to other antibiotic classes, except for carbapenems. Pyoverdine and pyocyanin production showed insignificant decreases, whereas there was a significant decrease in rhamnolipid production. A total of 1039 proteins were identified, of which approximately 25 % were DEPs. In general, there were more downregulated proteins than upregulated proteins. Noted changes included decreased OprD and PilP, and increased MexEF-OprN, MvaT and Vfr, as well as proteins of ribosome machinery and metabolism clusters. Gene expression analysis confirmed the proteomic data and indicated the downregulation of *rpoB* and *rpoS*. In summary, the response to CIP involved approximately a quarter of the proteome, primarily associated with ribosome machinery and metabolic processes. Potential targets for bacterial interference encompassed outer membrane proteins and global regulators, such as MvaT.

## Graphical Abstract

Sub-MICs of ciprofloxacin result in DNA breakage, triggering DNA damage stress and the SOS stress response. This, in turn, induces changes in regulatory factors and the translation machinery. Modifications to regulatory components, including MvaT, RpoS and RpoB, lead to the upregulation (depicted in green) of efflux pumps such as MexEF-OprN and the downregulation (depicted in red) of porins. Additionally, there are alterations in the expression of virulence factors. Concurrently, ciprofloxacin affects metabolic processes, shortens cell lengths and induces the formation of multinucleated filaments due to disruptions in cell replication.

## Data Summary

All the data are provided in the manuscript paper with its supplementary files and proteomic data are available on Figshare at https://doi.org/10.6084/m9.figshare.25140002 [1].

## Introduction

*Pseudomonas aeruginosa* is a Gram-negative, rod-shaped, asporogenous, monoflagellated bacterium boasting one of the largest genomes in comparison to bacteria such as *Escherichia coli* and *Staphylococcus aureus* [2,3]. This opportunistic pathogen is a prevalent cause of nosocomial infections, contributing to high morbidity and mortality rates [4]. In clinical settings, *P. aeruginosa* isolates have been documented to exhibit resistance to nearly all classes of commonly used antibiotics, including aminoglycosides, cephalosporins, fluoroquinolones and carbapenems [5].

Ciprofloxacin, a fourth-generation fluoroquinolone, is used to treat a wide range of *P. aeruginosa* infections. Ciprofloxacin was initially very effective, but *P. aeruginosa* responded rapidly and acquired high-level resistance [6]. The resistance mechanisms to fluoroquinolones mainly include target-site modifications which are mutations in gyrase and/or topoisomerase and upregulation of efflux pumps such as MexCD-OprJ and MexEF-OprN. Mutations in *gyrA, gyrB, mexA, mexB, nfxB* and *mex*Z are frequently found in fluoroquinolone-resistant clinical isolates [7,8].

Studies have suggested that sub-MICs of antibiotics, frequently observed in the environment, human body and tissues, especially during antibiotic therapy, due to low bioavailability or inadequate dosage can lead to the adaptive evolution of highly resistant isolates expressed via the change in their proteomes and phenotypes [9,10].

Previous work in *in vitro*-induced ciprofloxacin resistance has found that after 14 days of exposure to ciprofloxacin, *P. aeruginosa* strains developed resistance to ciprofloxacin with MIC values increased 32-fold [11]. The resistant phenotype and its altered virulence, including protease activity, biofilm formation and motility, did not revert fully after the exposed strain was cultured for 10 days in an antibiotic-free environment [11].

In this study, proteomic and phenotypic responses of *P. aeruginosa* to ciprofloxacin and markers associated with the development of antibiotic resistance were investigated. The markers found were expected to serve as additional targets to minimize fluoroquinolone resistance development, thus reviving this valuable antibiotic class for clinical application.

## Methods

### Bacterial strains

Strains CIP-E1 and CIP-E2 were generated from *P. aeruginosa* ATCC 9027 in a previous study (Fig. S1, available in the online version of this article) [11]. In brief, CIP-E1 was the 14 day ciprofloxacin-exposed *P. aeruginosa* ATCC 9027 and CIP-E2 was the 10 day antibiotic-free cultured CIP-E1. The length of 14 days was selected after repeated experiments with exposure until 20 days showing that for most antibiotics, after 12–14 days, the bacterium no longer increased its MIC under antibiotic pressure. The length of 10 days was selected as, at day 10, in most exposure replications, the bacterium maintained its MIC for at least three consecutive days. The antibiotic susceptibility profile determined via an agar- diffusion assay and virulence of the strains used in this study were reported and are provided in Tables S1, S2 and S3 [11]. All bacteria strains were stored at −80 °C in Tryptic Soya broth (TSB; HiMedia) with 30 % glycerol (TSB/glycerol 7 : 3, v/v). For each subsequent expriment, the samples were thawed directly from storage and cultured at 37 °C, overnight at 120 r.p.m. in TSB.

### Determination of MICs against frequently used antibiotics

A 96-well plate with concentration gradients of tested antibiotics, including ciprofloxacin, ofloxacin, levofloxacin, ceftazidime, meropenem, gentamicin and piperacillin/tazobactam, was prepared. Each well contained 100 µl of the antibiotic diluted by the standard twofold dilution series in Muller-Hinton broth (MHB; HiMedia). Overnight cultures of the three strains (*P. aeruginosa* ATCC 9027, CIP-E1 and CIP-E2) with an OD_600nm_ of approximately 0.08–0.1 were diluted 1 : 100, and 100 µl of the diluted cultures was inoculated into each well of the plate. The plate was then incubated at 37 °C for 18–24 h, and the MIC value was recorded. The experiment was duplicated.

### Phenotypic analysis

#### Cell morphology analysis using scanning electron microscopy

Samples were prepared according to a previous protocol [12]. Briefly, each strain was incubated with sterilized polypropylene stubs overnight at 37 °C. The stubs were fixed with 10 % formalin for 24 h and dried through an ethanol series, 30, 40, 50, 60, 70, 80, 90, 95 and 100 % before drying for 12 h in t-butyl alcohol. The fixed samples were coated with gold and observed under a scanning electron microscope (Coxem). Images captured were analysed by ImageJ (NHI) software, and 30 cells from each sample were measured and analysed using IBM SPSS Statistic 25 (IBM). To assess filament prevalence, distinct regions with separated individual cells were demarcated in scanning electron microscopy (SEM) images for each strain. The number of filaments and the total cell count were determined using the Cell Counter Plugin available at https://imagej.net/ij/plugins/cell-counter.html within the ImageJ (NHI) software. The percentage of filaments was computed as the ratio of the number of filaments to the total counted cells, multiplied by 100 %.

#### Evaluation of pyocyanin, pyoverdine and rhamnolipid production

Overnight cultures were evaluated for the production of rhamnolipids, pyocyanin and pyoverdine using previous protocols [13,15] with modifications.

For pyocyanin production, 100 µl of overnight culture (OD_600nm_ of 0.08–0.1) was inoculated in 5 ml of glycerol-alanine medium (1 % glycerol, 0.14 % MgCl_2_, 1 % K_2_SO_4_, 0.1 % d-alanine and 2 % peptone) overnight at 37 °C. Pyocyanin was extracted with chloroform and 0.2 M HCl and 125 µl of the red layer was transferred to a 96-well microtitre plate. The concentration of pyocyanin (μg µl^–1^) was estimated by multiplying the optical density (OD) at 520 nm by 17.072, following the previous study [16]. *E. coli* was used as a negative control.

For pyoverdine production, 100 µl of diluted culture (OD_600nm_ of 0.08–0.10) was inoculated in 5 ml of King B broth (HiMedia), and incubated overnight at 37 °C at 180 r.p.m. The supernatant was collected by centrifugation and filtration, and fluorescence was measured in a BioTek Synergy HTX Multimode Reader (Agilent) at an excitation and emission of 405 and 450 nm, respectively [15].

For rhamnolipid production, 10 µl of the overnight inoculum was added into each well of a Siegmund Wagner (SW) agar plate following the protocol of Pinzon *et al*. [14]. The plates were incubated for 48 h at 37 °C and then stored in the refrigerator for at least 24 h (typically 48 h). A fixed light source such as that of a dissecting microscope was used to illuminate the plates. ImageJ was used to determine the halo diameters produced after incubation.

Each assay was conducted in biological triplicates and analysed using MS Excel (Office 365; Microsoft).

### Proteomic analysis

Protein samples were prepared as previously decsribed [17]. In brief, *P. aeruginosa* ATCC 9027, CIP-E1 and CIP-E2 were cultured overnight in TSB (HiMedia). With CIP-E1, the ciprofloxacin MIC was double checked and ciprofloxacin was supplemented in culture at a concentration of 2 μg µl^–1^. Cell pellets were collected via centrifugation at 13000 r.p.m. for 30 min at 4 °C. Cell membranes were disrupted by sonication at 20–50 kHz in 10 s intervals for 5 min on ice and protein samples were collected via centrifugation at 13000 r.p.m. for 30 min at 4 °C. Each protein sample was reduced, digested with trypsin and labelled with iTRAQ Reagent Kit (AB SCIEX) using an eight-plex procedure. The iTRAQ-labelled mixture was desalted, purified and analysed by LC-MS/MS coupled with iTRAQ analysis. The experiment was duplicated with the sample from *P. aeruginosa* ATCC 9027 as the internal control.

### Gene expression analysis using quantitative qRT-PCR

Expression of genes coding for notable differentially expressed proteins (DEPs) and their related regulatory genes were verified and analysed by quantitative reverse transciptase PCR (qRT-PCR) [18]. Briefly, total RNA was isolated via an RNA isolation kit (New England Biolabs), and cDNA was produced using the SensiFast cDNA synthesis kit (Meridan/Bioline). Primers designed in this study were designed by primerBLAST, and checked with Primer3, verified by gradient PCR. qRT-PCR was conducted with SensiFast SYBR no-ROX (Meridan/Bioline) using primers (PHUSA; Table 1).

**Table 1.** Primers used in qRT-PCR

Gene	Sequence (5′−3′)(F: forward; R: reverse)	Size (bp)	Reference
*rpsL*	F- CGGCACTGCGTAAGGTATGCR- CGTACTTCGAACGACCCTGCT	212	[50]
*mvaT*	F- GCCTGAAGTCCCTGGAACAAR- TGCGGGTTCTTGTACTGCTT	208	[51]
*recA*	F- AGATCATCGATCTGGGCGTGR- AACTGGTCGCGAATGGTCTT	163	This study
*mexE*	F- TCATCCCACTTCTCCTGGCGCTACCR- CGTCCCACTCGTTCAGCGGTTGTTCGATG	150	[50]
*mexA*	F- GGCGACAACGCGGCGAAGGR- CCTTCTGCTTGACGCCTTCCTGC	230	[50]
*pilP*	F- CAGAAGGGCAACAAGGTGATR- TCTCCGTCAGGAACGATTTC	245	This study
*oprD*	F- GGGCCGTTGAAGTCGGAGTAR- GGCGACAACGCGGCGAAGG	194	[32]
*rpoB*	F- AGGAACCGCGGTAAGGAATGR- AATCCCCCTGATGACCGAGA	170	This study
*rpoD*	F- GGGCGAAGAAGGAAATGGTCR- CAGGTGGCGTAGGTGGAGAA	178	[52]
*rpoS*	F- CTCCCCGGGCAACTCCAAAAGR- CGATCATCCGCTTCCGACCAG	198	[52]

The experiment was performed in triplicate. Fold change and 95 % confidence interval (CI) were calculated in MS Excel (Office 365; Microsoft) [19,20].

### Data analysis

IBM SPSS Statistics 20.0 (IBM) was used to analyse the data. One-way ANOVA, followed by post-hoc Tukey honest signicant difference (HSD) test was used to determine the difference in morphology and virulence factor production of *P. aeruginosa* strains. The *P*-value or significance was set to <0.05.

Raw MS/MS data were analysed using ProteinPilot Software 4.5 (AB SCIEX). Proteins were identified using the Swiss-Prot/UniProt protein database. For protein identification, a threshold >0.05 (CI, 10 %) was applied with ProtScore at 2.0 and false discovery rate (FDR) at 1 %. DEPs were considered to be differentially expressed if their iTRAQ ratios were >1.5 (upregulation) or <0.667 (downregulation) in ciprofloxacin-exposed strains compared with the initial *P. aeruginosa* ATCC 9027.

A Venn diagram was constructed to analyse the common DEPs among exposed strains. Gene Ontology (GO) (PANTHER; Version 11.0, Protein Analysis Through Evolutionary Relationships; http://pantherdb.org) was used to evaluate the biological significance of the DEPs. Information on protein–protein interactions (PPIs) of the studied proteins was retrieved using the Search Tool for Retrieval of Interacting Genes/Proteins (STRING; http://string-db.org/).

## Results

### Changes in MICs of ciprofloxacin-exposed *P. aeruginosa*

The MIC for ciprofloxacin, ofloxacin and levofloxacin exhibited a significant increase in CIP-E1 and showed a partial reversal in CIP-E2. Both CIP-E1 and CIP-E2 retained their resistance to ciprofloxacin, ofloxacin and levofloxacin. Notably, CIP-E1 developed resistance to meropenem, but not to ceftazidime. Impressively, CIP-E1 and CIP-E2 demonstrated increased sensitivity to gentamicin (MIC=0.25 and 0.5 µg ml^−1^, respectively) compared to the initial strain, * P. aeruginosa* ATCC 9027 (MIC=2 µg ml^−1^) (Table 2).

**Table 2.** MICs (µg ml^−1^) of antibiotics against *Pseudomonas aeruginosa* ATCC 9027 and the ciprofloxacin-exposed strains Antibiotic susceptibility was evaluated according to CLSI 2020 Performance Standards for Antimicrobial Susceptibility Testing (R=resistance, S=susceptible, i=Intermediate). The result was representative of two replicates which were generally identical.

	ATCC 9027	CIP-E1	CIP-E2
Ciprofloxacin	0.125 (S)	**16 (R**)	**4 (R**)
Ofloxacin	1 (S)	**64 (R**)	**16 (R**)
Levofloxacin	0.5 (S)	**32 (R**)	**8 (R**)
Ceftazidime	1 (S)	0.5 (S)	1 (S)
Meropenem	2 (S)	**16 (R**)	2 (S)
Gentamicin	**2** (S)	**0.25** (S)	**0.5** (S)
Piperacillin/tazobactam	4 (S)	2 (S)	2 (S)

### Phenotypic alterations in ciprofloxacin-exposed *P. aeruginosa*

**Fig. 1.**
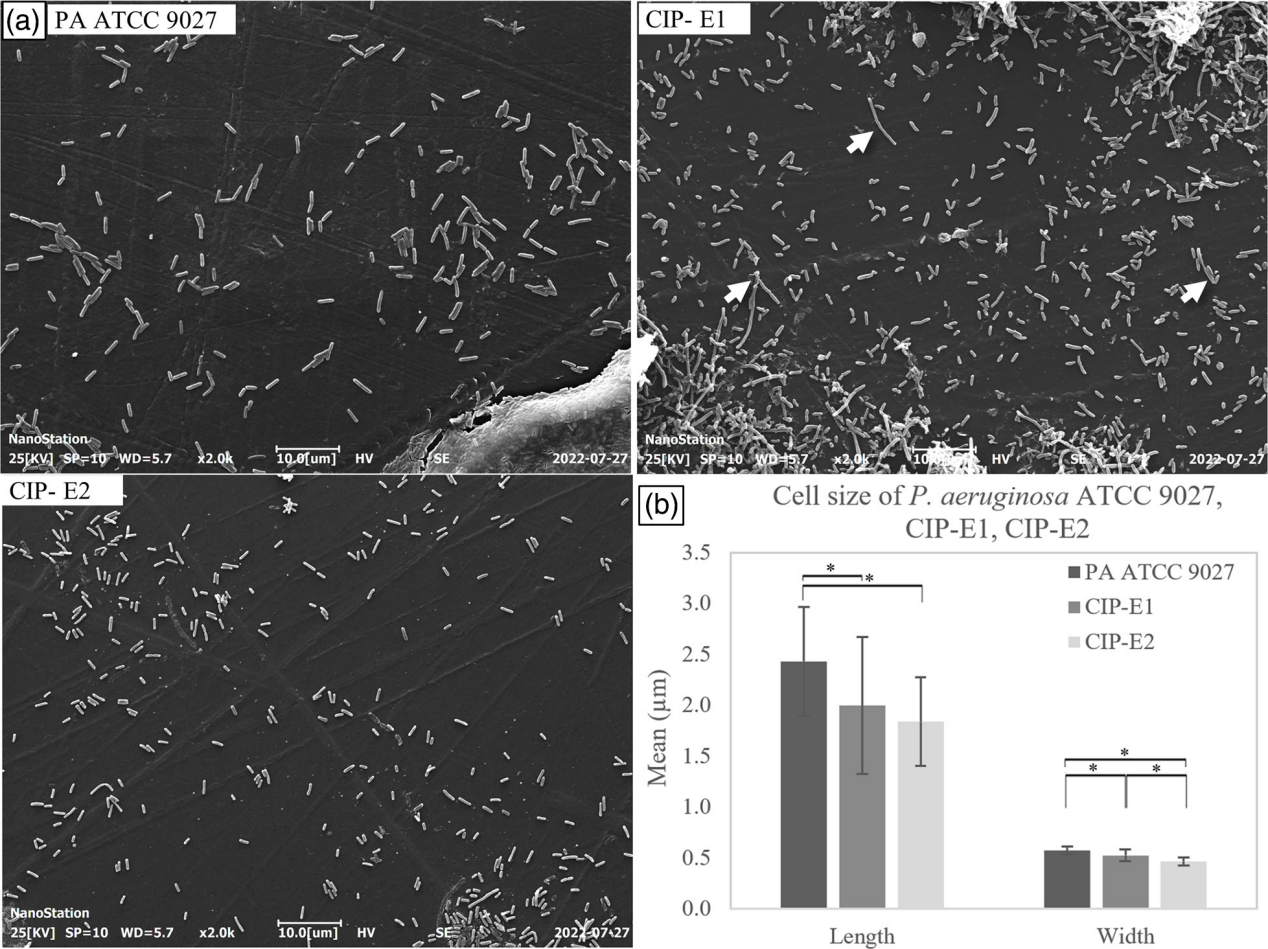
Morphological changes of *P. aeruginosa* in response to ciprofloxacin. (**a**) SEM images of the initial unexposed strain (*P. aeruginosa* ATCC 9027), the strain exposed to ciprofloxacin for 14 days (CIP-E1) and strain CIP-E1 after 10 days of antibiotic-free culture (CIP-E2). The bacterial culture was incubated with PPE stubs and then fixed with 10 % formalin for 24 h. The fixed samples were dried through serial concentrations of ethanol and coated with gold before the image was captured at 2000× magnification. (**b**) Cell length and width of *P. aeruginosa* ATCC 9027, CIP-E1 and CIP-E2. The length and width of 30 cells of each sample were measured by ImageJ, analysed by SPSS and presented as mean±sd. *Statistically significant difference (*P*<0.05).

**Fig. 2.**
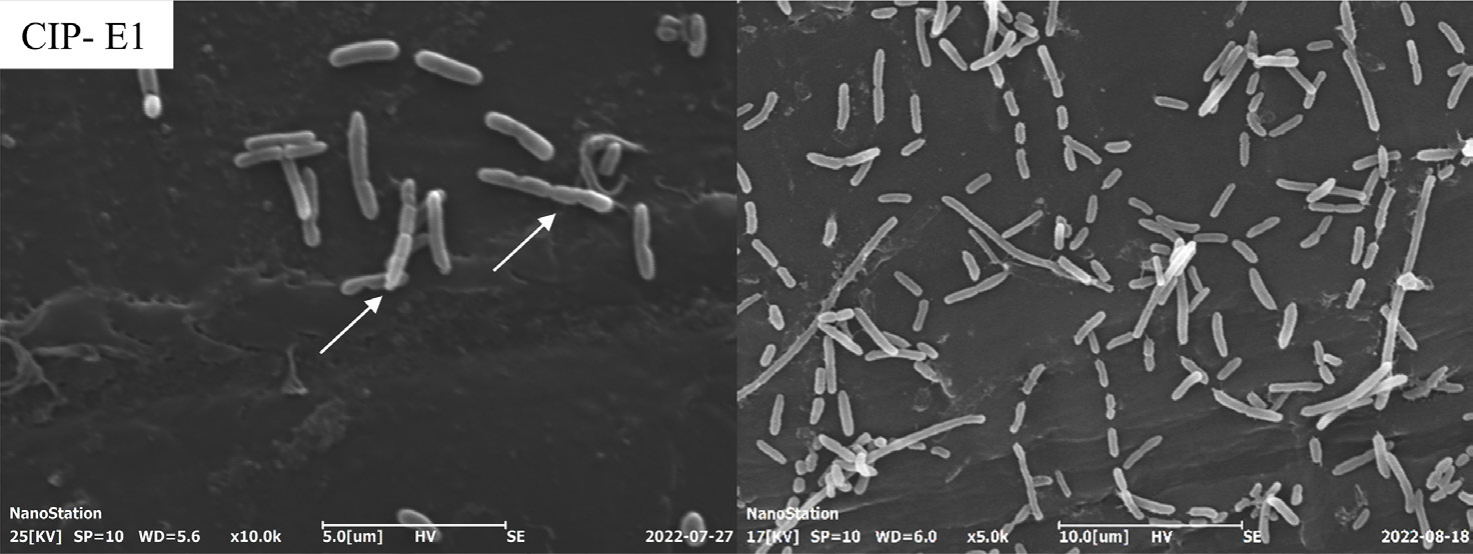
CIP-E1 showing filamented cells under SEM. The CIP-E1 sample was incubated with a PPE stub and then fixed with 10 % formalin for 24 h. The fixed samples were dried through serial concentrations of ethanol and coated with gold before the image was captured at 10 000× (left) and 5000× (right) magnification.

A significant reduction in mean length (*P*<0.05) was observed between CIP-E1 (1.99±0.67 µm) and CIP-E2 (1.84±0.43 µm) compared to *P. aeruginosa* ATCC 9027 (2.43±0.53 µm) (Fig. 1b). Additionally, CIP-E1 exhibited greater heterogeneity in length, as indicated by a larger standard deviation. Furthermore, there was a notable decrease in mean width (*P*<0.05) from 0.57±0.04 µm in *P. aeruginosa* ATCC 9027 to 0.53±0.06 µm in CIP-E1 and 0.47±0.04 µm in CIP-E2 (Fig. 1b). In CIP-E1, connected cells and cells arrested in the division state were clearly seen (Fig. 1a, CIP-E1, arrows, and Fig. 2). The prevalence of filamented cells in CIP-E1 was 2.60 %, whereas in CIP-E2, this was 0.42 %. Although cell arrest was no longer observed in CIP-E2, the reduced-size phenotype persisted (Fig. 1a, CIP-E2).

Production of pyoverdin and pyocyanin non-significantly decreased in CIP-E1 and CIP-E2, with a slight recovery in CIP-E2 for pyoverdin but not for pyocyanin. In contrast, rhamnolipid production was reduced in both CIP-E1 and CIP-E2 (*P*<0.05) (Table 3).

**Table 3.** Alterations of pyoverdin, pyocyanin and rhamnolipid production of *P. aeruginosa* under exposure to ciprofloxacin

	ATCC 9027	CIP-E1	CIP-E2
Pyoverdin (emission: 405, excitation: 450)	259.2±22.4	222.7±52	240.5±40.5
Pyocyanin (µg ml^−1^)	1.34±1.48	0.71±0.50	0.43±0.50
Rhamnolipid (mm)	12.63±0.15	11.33±0.26*	11.54±0.56*

* Significantly different from PA ATCC 9027 (*P*<0.05).

### Proteomic alterations in ciprofloxacin-exposed *P. aeruginosa*

**Fig. 3.**
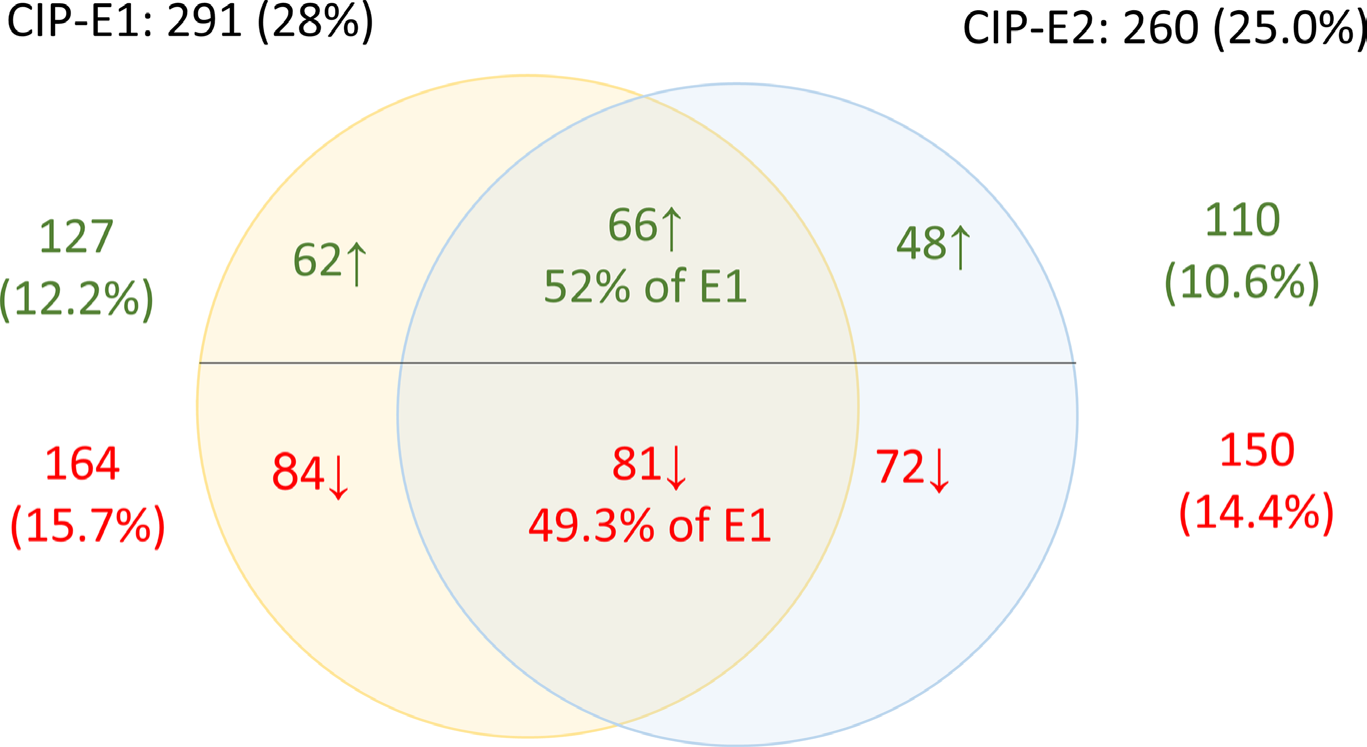
Differentially expressed proteins in ciprofloxacin-exposed *P. aeruginosa* strains. Differentially expressed proteins in CIP-E1 (yellow circle) and CIP-E2 (blue circle) include upregulated proteins (green; normalized fold change >1.5) and downregulated proteins (red; normalized fold change <−1.5). The number is expressed as *n* (% of total identified proteins) unless otherwise indicated.

A total of 1039 proteins were identified, of which approximately 25 % were DEPs, as summarized by a Venn diagram in Fig. 3. In general, there were more downregulated than upregulated proteins in both CIP-E1 and CIP-E2, compared to the initial * P. aeruginosa* ATCC 9027. In summary, 291 DEPs were identified in CIP-E1, including 164 downregulated and 127 upregulated proteins. In CIP-E2, there were only 260 DEPs, including 110 upregulated and 150 downregulated proteins. Approximately 52 % of the upregulated proteins and 49.3 % of the downregulated proteins in CIP-E1 maintained their expression change in CIP-E2 (Fig. 3). Note also that proteins of sample CIP-E1 were extracted when the bacteria was still cultured under the presence of ciprofloxacin at 2 µg ml^−1^ while samples from *P. aeruginosa* ATCC 9027 and CIP-E2 were not. A value of 2 µg ml^−1^ was chosen as double checking indicated that the MIC of CIP-E1 to ciprofloxacin remained consistently at 16 µg ml^−1^.

**Fig. 4.**
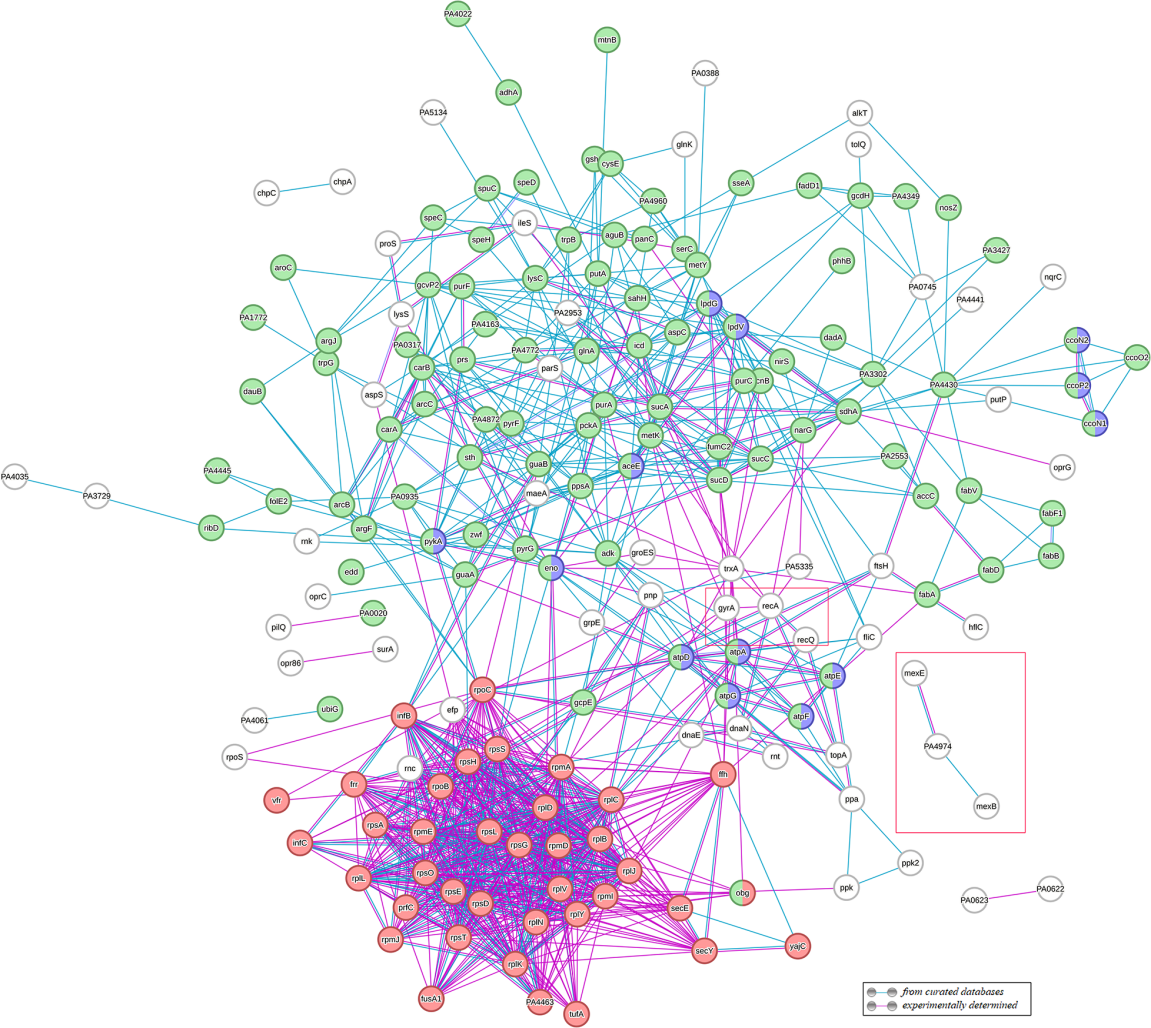
Protein–protein interaction network of differentially expressed proteins in CIP-E1 using the STRING database. Only known interactions from experimental evidence and existing databases are shown. Disconnected nodes are hidden (https://string-db.org/). Ribosome and translation regulators (red); metabolic pathways (green); ATP metabolic process (purple). Red boxed: efflux pumps and DNA recombination proteins. Protein–protein interactions are shown in evidence view and proteins were linked based on curated, experimental evidence. Network analysis was set at high stringency (STRING score=0.7).

Mapping DEPs using the STRING database resulted in a complicated protein–protein interaction network (Fig. 4). Two large clusters were the cluster of ribosomal proteins and their partners and the cluster of metabolic pathway proteins, especially those involved in modulating stimuli-responding metabolic changes. There were also chaperon proteins and stress response proteins, such as DNA repair proteins RecA and RecQ, chaperon proteins GroES, GrpE and ClpB, and chemotaxis proteins (ChpA, ChpC). Other ciprofloxacin-responding clusters included efflux pump proteins and porin proteins. All these DEPs were clearly linked and together they formed a network in response to fluoroquinolone exposure.

**Fig. 5.**
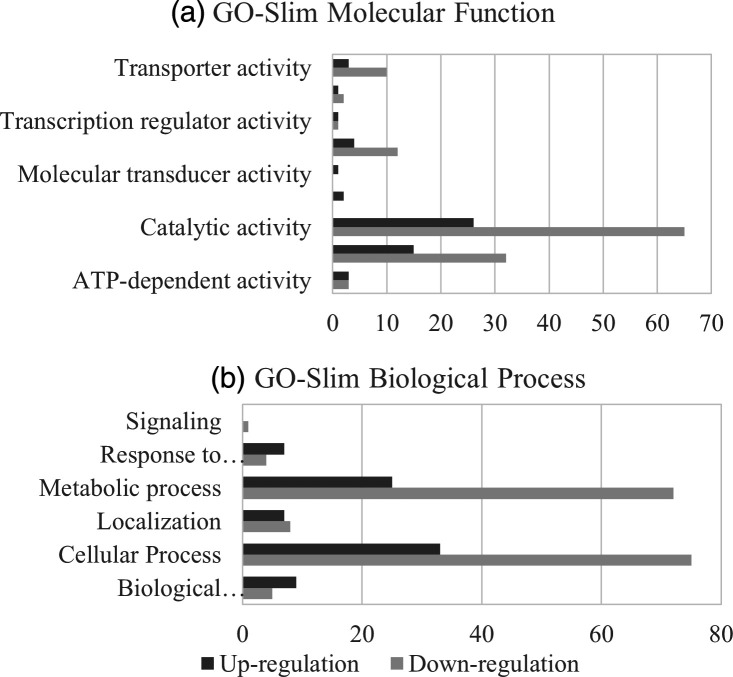
Summary of differentially expressed proteins in CIP-E1 by (**a**) GO-Slim molecular function and (**b**) GO-Slim biological process. Annotation of the differential expressed proteins was via the PANTHER database (http://pantherdb.org/).

GO annotations of the DEPs of CIP-E1 were classified into several categories and analysed using Panther software (Fig. 5). In the Molecular function categories, the majority of DEPs had catalytic activity and binding functions (Fig. 5a). Among Biological process, against the general trend for DEPs, there were more upregulated than downregulated proteins in the categories of response to stimulus and biological regulations, indicating a shift to adapt to the antibiotic stress (Fig. 5b).

**Fig. 6.**
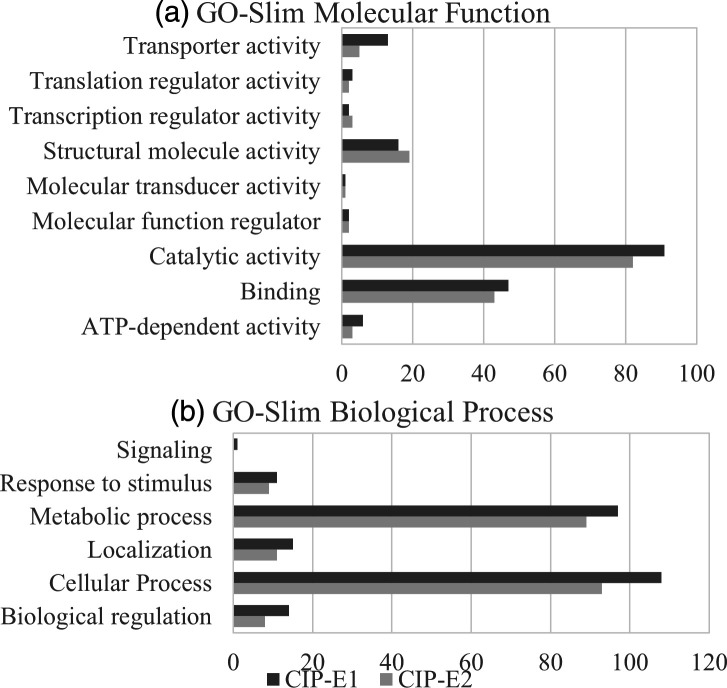
Summary of differentially expressed proteins in CIP-E1 and CIP-E2 using (**a**) GO-Slim molecular function and (**b**) GO-Slim biological process. Annotation of the differential expressed proteins was via the PANTHER database (http://pantherdb.org/).

Comparing DEPs of CIP-E1 and CIP-E2, similar profiles were observed in both strains. As expected, there were more DEPs in CIP-E1 than in CIP-E2. The largest difference was in proteins with transporter activity, which included efflux pumps and porins (Fig. 6).

Notable DEPs are summarized in Table 4. Gyrase A, a target of ciprofloxacin, was downregulated (to −1.60-fold in CIP-E1 and −1.96-fold in CIP-E2), while RecA and other proteins of DNA repair mechanisms were upregulated. Efflux pump proteins showed varying changes in response to ciprofloxacin exposure. MexE, MexF and OprN (efflux pump MexEF-oprN) and PA2812 (probable ATP-binding component of ABC transporter) were notably upregulated in CIP-E1 but not in CIP-E2 (Table 3), while MexA and MexB were downregulated. In addition, OprD was downregulated in both CIP-E1 and CIP-E2 (−17.21- and −1.46-fold, respectively). Expression levels of many global transcriptional regulators changed. Notably, Vfr and MvaT were significantly downregulated and upregulated, respectively, in both exposed strains. In addition, proteins involved in motility, the Type 4 pilus system, were downregulated.

**Table 4.** Selected differentially expressed proteins in CIP-E1 and CIP-E2 Proteins are grouped according to their functions and interactions. Fold change values are given for CIP-E1 and CIP-E2.

Accession no.	Name	Gene	CIP-E1	CIP-E2
GYRA_PSEAE	DNA gyrase subunit A	*gyrA*	−1.60	−1.96
RECA_PSEAE	Protein RecA	*recA*	3.60	−2.78
Q9HYQ1_PSEAE	DNA helicase	*recQ*	1.63	2.07
Q9I0Y9_PSEAE	Resistance-nodulation-cell division (RND) multidrug efflux membrane fusion protein MexE	*mexE*	4.45	−2.56
Q9I0Y8_PSEAE	Efflux pump membrane transporter	*mexF*	18.03	1.11
Q9I0Y7_PSEAE	Multidrug efflux outer membrane protein OprN	*oprN*	11.48	−3.57
Q9I031_PSEAE	Probable ATP-binding component of ABC transporter	PA2812	2.00	1.22
G3XD89_PSEAE	Putative copper transport outer membrane porin OprC	*oprC*	−14.33	−0.98
PORD_PSEAE	Porin D	*oprD*	−17.21	−1.46
G3XDA5_PSEAE	Anaerobically induced outer membrane porin OprE	*oprE*	−2.00	−1.27
PORF_PSEAE	Outer membrane porin F	*oprF*	−2.61	−1.04
Q9HW86_PSEAE	MvaT	*mvaT*	3.37	3.80
RPOS_PSEAE	RNA polymerase sigma factor RpoS	*rpoS*	1.87	1.64
VFR_PSEAE	cAMP-activated global transcriptional regulator Vfr	*vfr*	−2.88	−2.01
PILM_PSEAE	Type IV pilus inner membrane component PilM	*pilM*	−1.60	−1.46
PILP_PSEAE	Type IV pilus inner membrane component PilP	*pilP*	−1.94	−1.03
PILQ_PSEAE	Fimbrial assembly protein PilQ	*pilQ*	−1.61	−1.33

### Changes in gene expression in ciprofloxacin-exposed *P. aeruginosa*

**Fig. 7.**
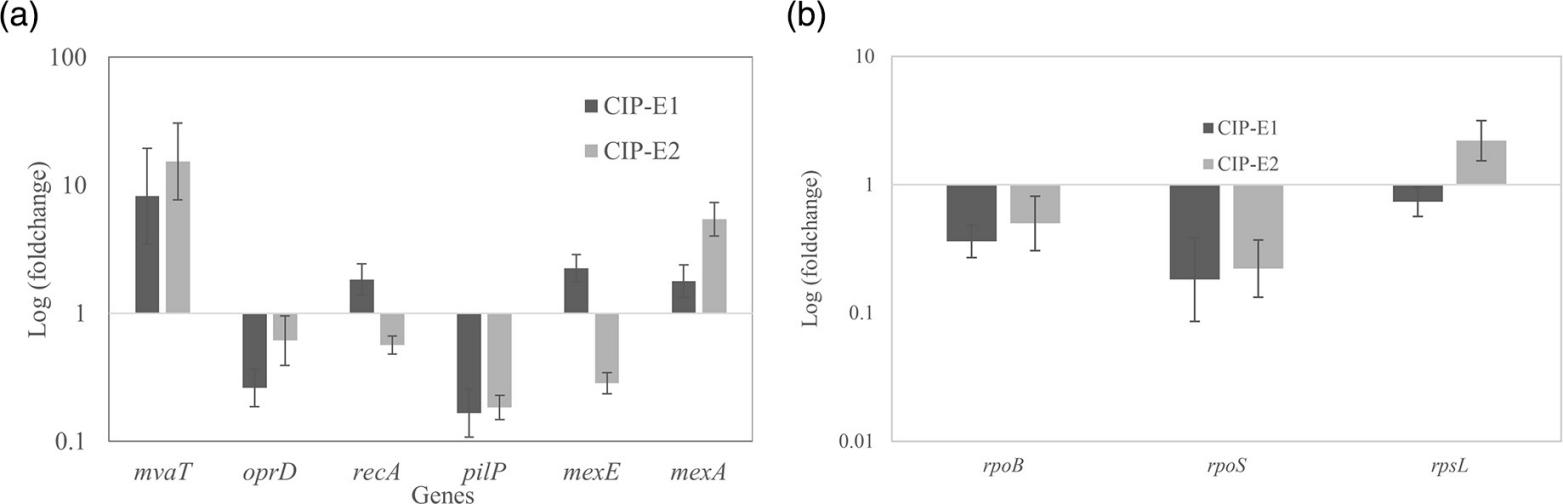
Gene expression alterations in *P. aeruginosa* and its ciprofloxacin-exposed strains. (**a**) Expression changes of selected genes from Table 4 and (b) expression changes of selected global regulators and transcription factors. Fold change was calculated against *P. aeruginosa* ATCC 9027 and visualized on a log scale, with gene expression of *P. aeruginosa* ATCC 9027 as 1. Values >1 indicate upregulated genes, while values <1 indicate downregulated genes. Expression of the housekeeping gene *rpoD* was used as the reference gene value [19,53]. Fold change and confidence level (95 % CI, error bar) were calculated in MS Excel according to standard practice [20].

qRT-PCR analysis generally confirmed the proteomic data (Fig. 7a, Table S3). Compared to *P. aeruginosa* ATCC 9027, there were decreases in *oprD* and *pilP* gene expression and an increase in *mvaT* gene expression in CIP-E1 and CIP-E2. By contrast, *mexE* and *recA* results showed a slight increase in CIP-E1 but a decrease in CIP-E2, and the *mexA* gene showed a general increasing trend from CIP-E1 to CIP-E2 (Fig. 7a).

Because of the altered expression of proteins in metabolic and cellular processes, the expression of regulatory genes *rpoS, rpoB* and *rpsL* were also analysed by qRT-PCR. Under the effect of ciprofloxacin exposure, *rpoB* and *rpoS* expression decreased by −2.76- and −5.49-fold, respectively, while *rpsL* expression decreased insignificantly by −1.36-fold (Fig. 7b).

The fold changes in gene expression determined by qRT-PCR were in line with the proteomic results, although not in perfect agreement. This discrepancy suggests that protein levels were influenced by both transcriptional and post-transcriptional regulators. Nevertheless, the overall trend of fold changes in gene expression mirrored the general increase or decrease observed in the proteomic profile.

## Discussion

### Cell size decreased and filaments were formed under exposure to ciprofloxacin

Long filaments were observed in CIP-E1 (Fig. 2), which have also been reported in studies related to ciprofloxacin exposure. For example, in *E. coli*, exposure to genotoxic ciprofloxacin triggered the SOS response and induced the formation of long multi-chromosome-containing filaments [21]. The filaments resulted in new buds that had increased resistance to ciprofloxacin, after just 24 h exposure to the antibiotic. The filaments reflected incomplete DNA replication before cell division which promoted DNA repair and, thus, accelerated recombination between multiple chromosomes. In our study, as expected, upregulation of stress response proteins was observed in CIP-E1. In addition, extensive filaments have also been seen in *Klebsiella pneumonia, E. coli, Pseudomonas* and *Acinetobacter* spp. in response to not only ciprofloxacin but also to β-lactam antibiotic stress [22]. Thus, the filaments formed in response to antibiotics could be the result of many processes where arrested cell division is the main outcome.

Besides the presence of long filaments, reduced cell length and width in response to ciprofloxacin were also seen in this study. This observation was previously reported in a biofilm population of *P. aeruginosa* exposed to ciprofloxacin for 24 h [23] and also in *P. aeruginosa* exposed to carbapenem [24].

### Ciprofloxacin-induced virulence alterations corresponded with gene expression alterations

In ciprofloxacin-exposed strains, there was a reduction in the production of rhamnolipid, and to a lesser extent in pyocyanin and pyoverdin. Additionally, motility, biofilm formation and protease production decreased, suggesting potential evolutionary trade-offs between developing resistance and maintaining virulence (Table S2).

We observed an increase in MexEF-OprN expression which correlated with the development of ciprofloxacin resistance and decreased virulence. This correlation has been reported in previous studies. For example, Vaillancourt *et al*. showed that the PAO1 Δ*mexEF* strain was more susceptible to ciprofloxacin and showed increased swarming, rhamnolipid production and lethality in a mouse infection model [25]. In a different study, the overproduction of either MexCD-OprJ or MexEF-OprN resulted in antibiotic resistance but was also linked to a decrease in transcription of the type III secretion system (T3SS) regulon, leading to lower extracellular enzyme activity [26]. The increase in efflux pump expression and decrease in virulence factor expression were also seen in meropenem- and ceftazidime-exposed *P. aeruginosa* [27].

### Upregulation of efflux pumps and downregulation of porins involved in ciprofloxacin resistance development

Two common ciprofloxacin resistance mechanisms are alterations in the ciprofloxacin target protein (GyrA) or upregulation of efflux pumps [28,29]. In this study, the increased gene expression and protein upregulation of efflux pumps corresponded to the development of ciprofloxacin resistance in CIP-E1. This may also be linked to the fact that CIP-E1 was cultured in a sub-MIC of ciprofloxacin, so this upregulation response was highly required to pump out the antibiotics. Moreover, the highly elevated ciprofloxacin MIC in CIP-E1, compared to CIP-E2, demonstrated a corresponding increase in the expression of MexEF-OprN, specifically in CIP-E1. However, CIP-E2 maintained the ciprofloxacin resistance phenotype (MIC=4 µg ml^−1^), suggesting that the resistance here was attained only partially through overexpression of efflux pumps. Besides MexEF-OprN, other efflux-related proteins can also participate such as NfxB, a repressor for the transcription of *mexCD-oprJ*, a multidrug efflux pump [30]. In clinical strains, MexCD-OprJ upregulation correlated with increased resistance to ciprofloxacin, cefepime and chloramphenicol but increased susceptibility to ticarcillin, aztreonam, imipenem and aminoglycosides [31].

Previous studies of clinical isolates have linked downregulation or loss of OprD to carbapenem resistance [32,33]. OprD facilitates the passive intake of antibiotics, and its absence can reduce the penetration of antibiotics into the cell. Here, in the presence of ciprofloxacin, the decreased expression of OprD contributed to limited ciprofloxacin intake, thus increasing resistance to both ciprofloxacin and carbapenem. In our study, we noted that the reversion of porin expression led to the restoration of carbapenem susceptibility, but not fluoroquinolones.

It has been demonstrated that overexpression of MexT, a transcription factor of the *mexEF-oprN* operon, leads to increased efflux pump expression and reduced OprD expression [34]. In CIP-E2, while expression of the MexEF-OprN efflux pump was reversed, OprD expression also exhibited partial reversal. Previous studies have reported similar observations in meropenem- and ceftazidime-exposed *P. aeruginosa*, where OprD downregulation was coupled with the upregulation of a different efflux pump system, MexAB-OprM. This underscores the antibiotic-specific nature of efflux pump regulations, while porins such as OprD reflect a broader antibiotic response in *P. aeruginosa* [27].

### Changes in global regulators indicate complex changes to metabolism, cellular processes and virulence

Global regulators and transcription factors that responded to ciprofloxacin included RpoS, RpoB, MvaT and Vfr. RpoS and RpoB are components of the transcription machinery and their altered expression affected enzyme production and growth of the pathogens. The sigma factor RpoS influences the production of extracellular alginate and exotoxin A, the formation of biofilms, and also the expression of more than 40 % of all quorum-controlled genes discovered through transcriptome analysis in *P. aeruginosa* [35]. The reduction in *rpoS* gene expression corresponded with reduced virulence in CIP-E1 and CIP-E2, similar to the result reported by Suh *et al*., where a *rpoS101::aacCI null* mutant had reduced virulence compared to its parent strain [36]. In another study, *rpoS* expression was repressed by ofloxacin, and overexpression of *rpoS* promoted ofloxacin tolerance in an *lasR* mutant [37]. In addition, *rpoS* was also important in other antibiotic tolerance mechanisms and environmental stresses (through quorum-sensing and biofilm) [38,39]. On the other hand, in adaptive antimicrobial resistance, mutations in the *rpoB* gene have been linked to resistance phenotypes in *P. aeruginosa* and *E. coli* [40,42], though the mechanism remains unclear. The observed reduction of *rpoS* and *rpoB* gene expression might thus underlie the alterations in virulence and the development of ciprofloxacin development.

MvaT, as a negative regulator of the *cupA*, *cupB,* and *cupC* clusters, inhibits biofilm formation [43]. Thus, the upregulation of MvaT observed in exposed strains explained the observed decreased biofilm formation (Table S2). However, while MvaT continued to be upregulated in CIP-E2, biofilm formation recovered, indicating the complex regulation of biofilm production.

Vfr, the cAMP-activated global transcriptional regulator protein, was downregulated in both CIP-E1 (−2.88) and CIP-E2 (−2.01) (Table S1). Vfr controls virulence gene expression by distinct cAMP-dependent and cAMP-independent mechanisms, positively regulates the production of type IV pili (T4P), a T3SS, and the las quorum-sensing system, and negatively regulates flagellar gene expression. The downregulation of Vfr was consistent with observed changes in motility and biofilm formation (Table S2), as well as the downregulation of PilP and T4P, and changes in the metabolic process cluster observed from proteomic and qRT-PCR data as well as previous studies [44].

In short, the interplay between these regulators might underlie the observed varying alterations of protease, elastase, pyoverdin, pyocyanin and rhamnolipid production. Alterations in these global regulators led to phenotypic changes and antibiotic resistance [45].

### The development of ciprofloxacin resistance is multifactorial and involves multiple changes

Besides the alteration of proteins related to efflux pumps, porins and global regulators, other changes were also noted. For example, the downregulation of GyrA was seen in CIP-exposed strains indicating a convenient resistant mechanism where the target of ciprofloxacin was simply reduced. On the other hand, the upregulation of RecA and other proteins of DNA repair mechanisms indicated the need for DNA repair and the adaptation to the increased DNA damage caused by ciprofloxacin. This also explained why the multi-cellular filaments appeared in our study when DNA replication was interfered with. Interestingly, in ciprofloxacin-exposed strains, AmpC, a beta-lactamase, was downregulated in contrast to its upregulation in meropenem- and ceftazidime-exposed *P. aeruginosa* [27].

In both ceftazidime- and meropenem-exposed strains, a combination of OprD downregulation, AmpC upregulation and specific efflux pump activation was observed, contributing to resistance against ceftazidime and carbapenems [27]. In ciprofloxacin-exposed strains, we observed the downregulation of both OprD and AmpC, resulting in resistance to ciprofloxacin and carbapenems, while maintaining susceptibility to ceftazidime (Table S2).

From this study, it is clear that multiple mechanisms, including the combination of OprD downregulation, efflux pump upregulation and alterations in global regulators, contributed to the resistance to ciprofloxacin. In the process of adaptation to ciprofloxacin, * P. aeruginosa* not only altered pathways that confer resistance but also altered its virulence and metabolic pathways. Many alterations were evolutionary trade-offs, or even collateral sensitivity [46], which can be exploited to develop new therapeutic strategies. As observed here, while CIP-E1 developed resistance to ciprofloxacin, it also showed increased sensitivity to gentamicin (Table S2). Many studies have explored efflux pump inhibitors as a possible strategy for postponing resistance development [47] or drugs targeting alternative sigma factors to reduce virulence [48]. There have been clinical trials for drugs targeting metabolic pathways that can reduce virulence and potentiate antibiotic activity against *P. aeruginosa* in cystic fibrosis patients [49]. The regulators and sigma factors discussed above and their effectors might be other potential targets to prevent antibiotic resistance and improve antibiotic efficacy. However, for a better understanding of the mechanisms and identifying efficient interference of antibiotic resistance development, genomics and functional genomics investigations are needed.

## Conclusions

In this study, our data confirmed that sub-MIC values of ciprofloxacin triggered the stress response mechanisms in *P. aeruginosa*, leading to alterations in gene expression, morphology and virulence factors of the pathogen. Regulatory factors and the translation machinery were also among the genes responding most strongly. Alterations in expression of regulatory components, such as MvaT, RpoS, and RpoB, could explain the upregulation of efflux pumps, MexEF-OprN, and downregulation of porins as well as alterations of virulence factor expressions, which lead to the observed phenotypic changes and antibiotic resistance. Therefore, these regulators could serve as potential targets to mitigate the development of antibiotic resistance, thereby restoring the clinical applicability of ciprofloxacin. Moreover, mutations associated with the development of ciprofloxacin resistance in *P. aeruginosa* require in-depth investigation. Such research may provide a deeper understanding of the mechanisms behind resistance development, unveiling additional targets for intervention in the process.

## supplementary material

10.1099/mic.0.001443 Uncited Supplementary Material 1.

## Abbreviations

ATCC 9027: *P. aeruginosa* ATCC 9027
ATCC: american type culture collection
CI: confidence interval
CIP-E1: *P. aeruginosa* ATCC 9027 after 14 days in sub-MIC levels of ciprofloxacin
CIP-E2: CIP-E1 cultured in antibiotic-free media for 10 days
DEP: differentially expressed protein
qRT-PCR: quantitative reverse transcriptase PCR
T4P: type IV pili
T3SS: type III secretion system
