# Supplementary material for: Adaptive response of Pseudomonas aeruginosa under serial ciprofloxacin exposure
Source: Microbiology (Reading). 2024 Apr 3;170(3):001443. doi: 10.1099/mic.0.001443 (PMC11084610; doi:10.1099/mic.0.001443)
Supplement: Uncited Supplementary Material 1. [file mic-170-01443-s001.pdf]

Supplement materials

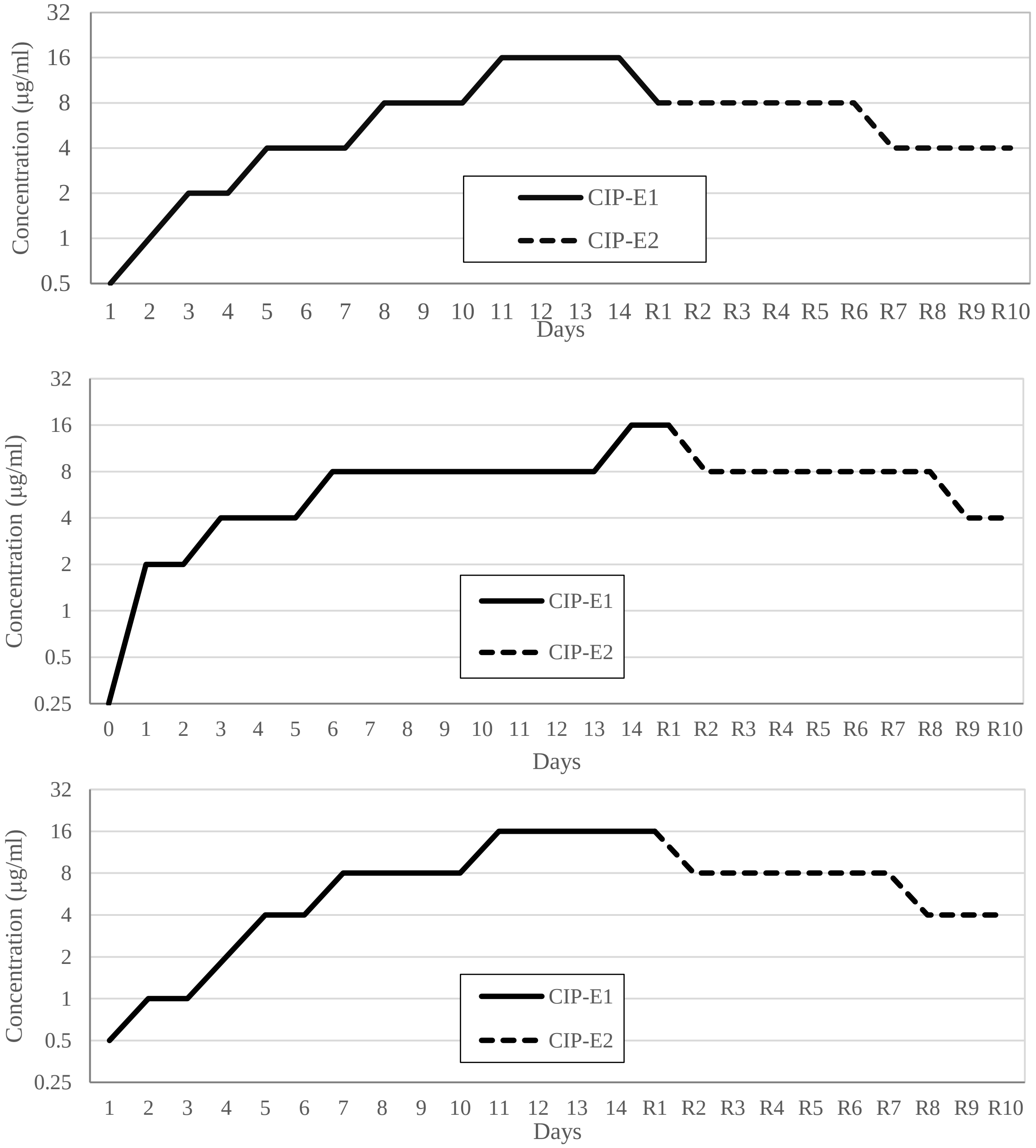

Figure S1. Changes of ciprofloxacin MIC values of *P. aeruginosa* during ciprofloxacin sub-MIC exposure. *P. aeruginosa* ATCC 9027 (MIC 0.5 µg/mL) was exposed to sub-MIC concentrations of ciprofloxacin (Nam Khoa Biotek Co., Ltd.) for 14 days to obtain CIP-E1 (MIC 16.0 µg/mL), then cultured in antibiotic-free environments for 10 days to obtain CIP-E2 (MIC 4 µg/mL). R1-R10 denoted the days of culturing in an antibiotic-free medium. Three replicates were presented.

Table S1. Antibiotic susceptibility profile of *Pseudomonas aeruginosa* ATCC 9027 and exposed strains.

Antibiotic susceptibility was evaluated according to CLSI 2020 Performance Standards for Antimicrobial Susceptibility Testing (R=Resistance, S=Susceptible, I=Intermediate).

	PA ATCC 9027	CIP-E1	CIP-E2
**Ciprofloxacin**	**S**	**R**	**R**
**Levofloxacin**	**S**	**R**	**R**
**Ofloxacin**	**S**	**R**	**R**
Ceftazidime	S	S	S
Cefepime	S	S	S
Meropenem	S	S	S
Imipenem	S	S	S
Piperacillin/tazobactam	S	S	S
Tobramycin	S	S	S
Gentamicin	S	S	S

Table S2. Minimum inhibitory concentration of antibiotics against *Pseudomonas aeruginosa* ATCC 9027 and exposed strains.

Antibiotic susceptibility was evaluated according to CLSI 2020 Performance Standards for Antimicrobial Susceptibility Testing (R=Resistance, S=Susceptible, I=Intermediate).

	**REP 1**			**REP 2**		
µg/ml	**PA ATCC 9027**	**CIP-E1**	**CIP-E2**	**PA ATCC 9027**	**CIP-E1**	**CIP-E2**
Ciprofloxacin	0.125 (S)	**16 (R)**	**4 (R)**	0.25(S)	8**(R)**	4**(R)**
Ofloxacin	1 (S)	**64 (R)**	**16 (R)**	1(S)	32**(R)**	16**(R)**
Levofloxacin	0.5 (S)	**32 (R)**	**8 (R)**	1(S)	16**(R)**	8**(R)**
Ceftazidime	1 (S)	0.5 (S)	1 (S)	2(S)	0.5(S)	2(S)
Meropenem	2 (S)	**16 (R)**	2 (S)	4(S)	16(S)	4(S)
Gentamicin	**2** (S)	**0.25** (S)	**0.5** (S)	1 (S)	0.25 (S)	1 (S)
Piperacillin/tazobactam	4 (S)	2 (S)	2 (S)	4(S)	2(S)	2(S)

Table S3. Alterations of virulence factors of *P. aeruginosa* over exposure to Ciprofloxacin.

	PA ATCC 9027	CIP-E1	CIP-E2
Biofilm (OD_{550nm})	0.24±0.03	0.15±0.02	0.24±0.04
Protease (mm)	2.25±0.25	1.31±0.32	2.50±0.47
Elastin (mm)	4.68±0.62	2.55±0.33	4.2±0.37
Swimming	+++	+	+
Swarming	+++	+	++

characteristics measured on a relative scale of (-) no evidence of that phenotype; (+) low, (++) intermediate and (+++) high.

Table S4. Comparison of normalized protein expression fold change and mRNA fold change of exposed strains CIP-E1 and CIP-E2 against *P. aeruginosa* ATCC 9027.

	PROTEIN CHANGE		qPCR fold change	
	CIP-E1	CIP-E2	CIP-E1	CIP-E2
mvaT	3.37	3.8	3.15	11.16
oprD	-17.2	-1.46	-5.99	-2.24
recA	3.6	-2.78	1.18	-2.43
pilP	-1.94	-1.03	-9.43	-7.50
mexE	4.45	-2.56	1.44	-4.85
mexA	1.00	3.10	1.14	3.95
rpoB	-2.07	-1.94	-2.76	-2.00
rpsL	-1.75	-1.22	-1.36	2.20
rpoS	1.87	-1.68	-5.49	-4.51
